# Short-term Mobility and Increased Partnership Concurrency among Men in Zimbabwe

**DOI:** 10.1371/journal.pone.0066342

**Published:** 2013-06-18

**Authors:** Susan Cassels, Lisa Manhart, Samuel M. Jenness, Martina Morris

**Affiliations:** 1 Department of Epidemiology, University of Washington, Seattle, Washington, United States of America; 2 Department of Global Health, University of Washington, Seattle, Washington, United States of America; 3 Department of Sociology, University of Washington, Seattle, Washington, United States of America; 4 Department of Statistics, University of Washington, Seattle, Washington, United States of America; Vanderbilt University, United States of America

## Abstract

**Background:**

Migration has long been understood as an underlying factor for HIV transmission, and sexual partner concurrency has been increasingly studied as an important component of HIV transmission dynamics. However, less work has examined the role of short-term mobility in sexual partner concurrency using a network approach. Short-term mobility may be a risk for HIV for the migrant’s partner as well either through the partner’s risk behaviors while the migrant is away, such as the partner having additional partners, or via exposure to the return migrant.

**Methods:**

Using data from the 2010–11 Zimbabwe Demographic and Health Survey, weighted generalized linear regression models were used to investigate the associations between short-term mobility and partnership concurrency at the individual and partnership levels.

**Results:**

At the individual level, we find strong evidence of an association between short-term mobility and concurrency. Men who traveled were more likely to have concurrent partnerships compared to men who did not travel and the relationship was non-linear: each trip was associated with a 2% higher probability of concurrency, with a diminishing risk at 60 trips (*p*<0.001). At the partnership level, short-term mobility by the male only or both partners was associated with male concurrency. Couples in which the female only traveled exhibited less male concurrency.

**Conclusions:**

Short-term mobility has the ability to impact population-level transmission dynamics by facilitating partnership concurrency and thus onward HIV transmission. Short-term migrants may be an important population to target for HIV testing, treatment, or social and behavioral interventions to prevent the spread of HIV.

## Introduction

Migration, or the movement of persons from one country or locality to another, has long been understood as an underlying factor for transmission of sexually transmitted infections (STI), including HIV. Migration is thought to play a role in the spread of HIV in several countries with large generalized epidemics, including Kenya [1], South Africa [2], Uganda [3], and Zimbabwe [4]. Not only are migrants more likely than non-migrants to acquire HIV, but they are also more likely to subsequently transmit HIV to others [5]–[8]. For example, circular migrants, those who periodically travel to different geographic areas and back for work and other reasons, can influence HIV incidence in their home region by engaging in sexual risks and becoming infected with HIV while away and then infecting their main partners upon returning home. These associations may depend on the sex of the migrant as well, given potential differences in behavior and travel characteristics. Nonetheless, the role of frequent short-term mobility in generalized epidemics is less clear, and the link between short-term mobility and HIV could be confounded by reasons for travel [4], the geographic source population of migrants [9], or the stage of HIV epidemic [10]–[12]. Additionally, short-term mobility can be defined in many different ways; more work is needed to understand the nuanced relationship between sexual risk behavior and the duration and frequency of travel.

Concurrency, defined as overlapping sexual partnerships in which sexual intercourse with one partner occurs between two acts of intercourse with another partner, and other sexual network features such as assortative mixing (choosing demographically similar partners), can sustain high levels of HIV transmission in populations [13]–[17]. Migration may facilitate HIV transmission indirectly through concurrent sexual partnerships. However network-based research has largely ignored migration, particularly circular migration, as a dyadic attribute of sexual partnerships [18], even though travel may mediate the periodicity of multiple sexual partnerships, an essential building block for concurrency [19]. Circular migration can also be a risk for HIV for a migrant’s partner either through the partner’s risk behaviors while the migrant is away, such as the partner having additional partners, or via exposure to the return migrant. Network-based analysis is necessary to examine the patterns of travel and concurrency within partnerships [20]–[22].

The potential for short-term mobility to impact HIV transmission is particularly strong in Zimbabwe. The country has a relatively high-quality transportation infrastructure and diverse forms of movement including international travel and trade, migrant labor, and long- and short-term mobility driven by marriage and employment [23]. In one study in rural Zimbabwe, nearly one-third of adults were away from their home for more than one month in the prior year, with men and younger persons traveling most frequently [4]. There have been no population-based studies on the rates of and demographic factors associated with travel for the entire country. Zimbabwe also continues to have a severe HIV epidemic: national HIV prevalence was 15% in 2011 [24], [25], but 40% in sexually active young women [26]. Lastly, evidence from both empirical and modeling studies suggests that concurrent partnerships, even at low levels, lead to increased HIV in Zimbabwe and other countries in the region [14], [27]. Our study builds upon previous work on migration and HIV in Zimbabwe [4], [12], [28], [29] to address associations between short-term mobility and concurrency at the individual and partnership levels using data from the 2010–11 Zimbabwe Demographic and Health Survey (ZDHS).

## Methods

### Conceptual Framework

Migrants generally have higher levels of sexual risk behavior compared to non-migrants [30], especially in Africa [1], [2], [23]. Persons with a history of migration exhibit riskier behavior (suicide [31], drug use [32], crime [33], and early sexual debut [34]) compared to those who had not migrated. However, the association between mobility and disease are complex and nuanced, with recent research finding mixed evidence for the link [9], [10]. Migration theory suggests that the relationship depends on the type, distance, destination, duration, social context, and reason to move: whether the migration was “pushed” (migration motivated by leaving the origin) or “pulled” (migration for opportunity at the destination). Theoretically, persons choosing to migrate are positively selected on a number of human capital attributes, with those less able, motivated, or skilled less likely to migrate [35]. Positive selectivity is greater among migrants traveling longer than shorter distances [36]. Migrants may also be selected on sexual risk profiles as well; those that migrate may be more likely to take risks, or the ability to migrate may be associated with other predictors of risk such as wealth and opportunity. We adopt a conceptual framework, broadly called “risk propensity”, which combines selection and enabling affects: mobile persons are of a generally higher risk profile, and episodes of travel enables them to engage in that risk [37]. Migration and network epidemiology theory inform the model, which frames our specific hypotheses: 1) mobile men will exhibit higher concurrency than non-mobile men, but with a certain threshold for risk at the highest levels of travel; and 2) this association will also persist in dyadic analyses for the traveling partner, but the relationship may be weaker for the non-traveling partner due to a weaker selection effect.

### Procedures

The 2010–11 ZDHS, the fifth in a series of national-level population and health studies conducted as part of the global DHS program, was designed to provide data to monitor the public health of Zimbabwe, and follows-up on the 1988, 1994, 1999, and 2005–06 ZDHS studies. The 2010–11 study design has been described in detail [25], [38]. Briefly, the study utilized a two-stage sampling design, with frames based on the 2002 population census. First, 406 enumeration areas (169 urban and 237 rural) throughout Zimbabwe were sampled; second, 10,828 households within those areas were sampled. Data collection took place from September 2010 to March 2011. Study procedures included a standardized structured survey administered by a trained interviewer and an HIV test collected through dried blood spot filter paper. Consent was obtained before the survey and HIV specimen collection. All DHS datasets, including the 2010–11 Zimbabwe survey, are available online at the Measure DHS website. The data are publically available and anonymous, thus this study did not require IRB approval.

In the 10,828 selected households, 9,831 women aged 15–49 and 8,723 men aged 15–54 were eligible for the study. Of these, 9,171 women and 7,480 men participated and were interviewed. Among interviewees, 7,313 women and 6,250 men were tested for HIV. Of participants tested for HIV, 5,321 women and 3,843 men reported they were sexually active in the past year and aged 15 to 49; this subset was used for individual-level analyses. Of this group, there were 2,188 linked couples with complete data on HIV serostatus. The definition of and methods for recruiting these long-term cohabiting couples have been described elsewhere [25]. Of these couples, 1,852 had been together for at least 12 months (ensuring that concurrency and migration events in the last 12 months occurred while the couple was together); this subset, referred to as stable cohabiting couples, was used for dyadic analyses.

### Measures

Subjects were asked a variety of demographic and health-related questions. For this analysis, we considered age, education (no education, primary, secondary, higher), religion (Christian, Muslim, other/none), marital status (never, currently, formerly), sexual behaviors (sexual debut before age 15, multiple partners in last year, and for men only, male circumcision and ever paid for sex), and HIV status.

Short-term mobility was measured with two questions: “In the last 12 months, on how many separate occasions have you traveled away from your home community and slept away?”; and “In the last 12 months, have you been away from your home community for more than a month at a time?” For the individual-level analyses we measure short-term mobility as a continuous variable (number of trips) as well as travel-squared, since we hypothesized that the relationships between the frequency of travel and risk of concurrency is not linear. For the dyad-level analyses, we use the following discrete measures of past-year short-term mobility: any travel (1 or more overnight trips), frequent travel (10 or more trips), and long-duration travel (one or more trips lasting at least one month).

Sexual partnership concurrency was our main outcome measure. Twelve-month cumulative concurrency was measured using the duration (“For how long have you had a sexual relationship with this person?”) and date of last sex (“When was the last time you had sexual intercourse with this person?”) of the last three sexual partnerships. If there were any partnerships overlapping in time within the past year, the subject was categorized as having concurrent partners [39]. We additionally distinguished polygamous concurrency, since it may impact HIV transmission differently [40]. Thus, men’s concurrency was categorized as no concurrency, non-polygamous concurrency only, any polygamous concurrency. We do not show results for women’s concurrency due to small sample size and lack of significant variation.

### Statistical Analysis

Analyses accounted for the complex design structure of the study, with estimates weighted to adjust for unequal selection probability and non-response. The standard DHS weights for individual survey data, HIV testing data, and dyad data were used for this purpose, as described previously [25]. Variance estimates were based on the sampling dependence from the clustered design described above. Chi-square tests were used to determine statistically significant differences in the distribution of categorical variables by sex, and a rank-sum test was used to compare number of trips.

Multinomial logistic regression models estimated the relationship between travel and partnership concurrency among men. Multivariate models were constructed by incorporating sociodemographic and sexual behavior variables as covariates with the main exposure variable. The outcomes in the multinomial models were polygamous and non-polygamous concurrency, and the exposure was overnight travel. For summary and visualization purposes, we also combined both forms of concurrency. For the exploratory dyadic analysis, a contingency table of couples’ mobility status and partnership concurrency was constructed. Crude relative risks were calculated for all comparisons. Statistical analysis was conducted in Stata 12 (StataCorp, College Station, TX) and R 2.15 (R Foundation, Vienna, Austria).

## Results

Short-term mobility was common among both men and women, with 60.7% taking at least one overnight trip, and the average individual taking 4.8 trips in the past year. The number of trips was left-skewed overall, with a mean of 4.9, median of 1, minimum of 0, and maximum of 90 trips. Although the median was 1 trip for both men and women, men had a significantly higher distribution (*p*<0.001). Men were less likely than women to have traveled at all in the last year (59.1% vs. 62.1%, *p*  = 0.04), but more likely to travel 10 or more times (17.5% vs. 10.1%, *p*<0.001). Overall, 6.8% of men reported a concurrent partner in the last 12 months: 4.6% reported non-polygamous concurrency only, and 2.2% reported any polygamous concurrency. Less than 1% of women reported concurrent partnerships in the past 12 months. HIV prevalence was high overall, but significantly lower among men (15.7% vs. 18.3%, *p*<0.001), as shown in Table 1.

**Table 1:** Descriptive Characteristics of the 2010–11 Zimbabwe DHS: Men (n = 3,834) and Women (n = 5,321) Aged 15–49, Sexually Active in the Past Year, and Tested for HIV.

	Total		Men		Women		
	%	95% CI	%	95% CI	%	95% CI	*p*
**Sociodemographics**							
Age (mean)	30.2	30.0–30.5	31.1	30.8–31.4	29.5	29.3–29.8	<0.001
Education							<0.001
None	1.6	1.3–2.0	0.8	0.05–1.2	2.3	1.8–2.9	
Primary	28.7	27.0–30.4	23.3	21.3–25.4	33.2	31.4–35.1	
Secondary	64.1	62.4–65.9	68.4	66.2–70.5	60.5	58.6–62.4	
Higher	5.6	4.7–6.7	7.6	6.3–9.1	4.0	3.2–5.0	
Religion							<0.001
Christian	75.9	74.4–77.3	66.6	64.1–68.9	83.8	82.3–85.2	
Muslim	7.5	6.7–8.3	6.8	5.8–7.9	8.1	7.1–9.1	
Other/None	16.6	15.4–17.9	26.7	24.6–28.9	8.2	7.1–9.3	
Rural/Urban							0.37
Rural	71.2	68.8–73.4	70.7	67.9–73.3	71.6	69.2–73.8	
Urban	28.9	26.6–31.2	29.3	26.7–32.1	28.4	26.2–30.8	
Marital Status							<0.001
Never Married	13.1	12.1–14.1	21.8	20.3–23.5	5.7	4.9–6.5	
Currently Married	80.0	78.8–81.2	73.4	71.7–75.1	85.6	84.3–86.7	
Formerly Married	6.9	6.3–7.6	4.7	4.0–5.6	8.8	7.9–9.7	
**Sexual Behavior and HIV status**							
HIV-Infected	17.1	16.0–18.2	15.7	14.4–17.1	18.3	16.9–19.7	<0.001
Multiple Sexual Partners^2^	7.8	7.1–8.6	15.2	13.8–16.7	1.6	1.3–2.0	<0.001
Age at First Sex <15 Years	4.7	4.2–5.2	3.6	3.0–4.2	5.6	4.9–6.4	<0.001
Ever Paid for Sex	–	–	4.4	3.6–5.2	–	–	–
Ever Circumcised	–	–	10.1	8.9–11.5	–	–	–
**Short-term mobility**							
Overnight Travel^1^							
Number of trips (mean)	4.8	4.5–5.1	6.3	5.7–6.9	3.6	3.3–3.9	<0.001
≥1 Trips	60.7	59.3–62.1	59.1	56.8–61.3	62.1	60.3–63.8	0.04
≥10 Trips	13.5	12.7–14.4	17.5	16.1–19.1	10.1	9.2–11.1	<0.001
>1 Month Away	17.1	16.1–18.2	17.5	15.9–19.2	16.8	15.6–18.0	0.48
**Outcome**							
Concurrency^2^							<0.001
No	96.7	96.2–97.2	93.2	92.1–94.2	99.7	99.5–99.8	
Yes, Non-Polygamous	2.3	1.92–2.7	4.6	3.8–5.6	0.3	0.2–0.5	
Yes, Polygamous	1.0	0.8–1.3	2.2	1.7–2.8	0.0	–	

1 Analyses weighted with standard DHS sampling weights for study design and non-response.

2 In the past 12 months.

In bivariate analyses of factors associated with partnership concurrency for men (Table 2), higher education and ever paying for sex was associated with non-polygamous concurrency (RR  = 2.10; 95% CI  = 1.28–3.44; RR  = 5.65; 95% CI  = 2.82–11.29) but not with polygamous concurrency. Mobility, as number of trips, was positively associated with both non-polygamous and polygamous concurrency; each additional trip was associated with 2% higher risk of a concurrent partnership (RR  = 1.02; 95% CI  = 1.01–1.02; RR = 1.02; 95% CI: 1.01–1.03). Indeed, any travel and frequent travel were also significantly associated with increased concurrency, however long-duration travel was not independently associated with concurrency.

**Table 2:** Factors Associated with Sexual Partner Concurrency in the 2010–11 Zimbabwe DHS: Men (n = 3843) Aged 15–49, Sexually Active in the Past Year, and Tested for HIV (Reference: No Concurrency)^1^.

	Non-Polygamous Concurrency				Polygamous Concurrency			
	RR	95% CI	ARR^2^	95% CI	RR	95% CI	ARR^2^	95% CI
**Sociodemographics**								
Age (continuous)	1.01	0.99–1.02	1.02	1.00–1.03	1.07	1.04–1.09	1.07	1.04–1.10
Education: ≥ Secondary	2.10	1.28–3.44	1.89	1.14–3.10	0.91	0.54–1.55	1.05	0.59–1.87
Religion: Christian	0.89	0.60–1.32	0.81	0.54–1.22	1.05	0.64–1.74	0.97	0.58–1.63
Live in urban area	1.48	0.98–2.26	1.17	0.78–1.75	0.23	0.10–0.58	0.19	0.08–0.49
**Sexual Behavior**								
Age at First Sex <15 Years	1.27	0.58–2.76	1.32	0.78–1.75	0.41	0.08–1.87	0.56	0.13–2.34
Ever Paid for Sex	5.65	2.82–11.29	5.27	0.57–3.10	0.48	0.07–3.43	0.64	0.08–4.95
**Short-term mobility**								
Overnight Travel^3^								
≥1 Trips	2.29	1.55–3.41			1.90	1.10–3.29		
≥10 Trips	2.37	1.69–3.32			1.60	0.88–2.92		
>1 Month Away	1.27	0.80–2.02			0.92	0.47–1.82		
Trips (continuous)	1.02	1.01–1.02	1.05	1.02–1.08	1.02	1.01–1.03	1.04	1.00–1.08
Trips Squared	–	–	0.99	0.99–1.00				
Trips Squared & >1 Month (interaction)	–	–	1.00	1.00–1.00	–	–	1.00	1.00–1.00

1 Analyses weighted with standard DHS sampling weights for study design and non-response.

2 Adjusted for 4 sociodemographic and 2 sexual behavior variables in the table.

3 In the past 12 months.

In the multivariate model adjusting for potential confounders, we estimated the potential decline in effect of short-term mobility with increasing numbers of trips, along with the differential effects of long-duration travel, by modeling the probability of concurrency as a function of the interaction of travel-squared and long travel. The interaction term was not significant in the model (*p*  = 0.73), indicating no evidence of differential effect by travel duration. However, in a likelihood ratio test of nested models, there was strong evidence that the short-term mobility variables collectively explained the higher risk of concurrency (*p*<0.001).

**Figure 1:**
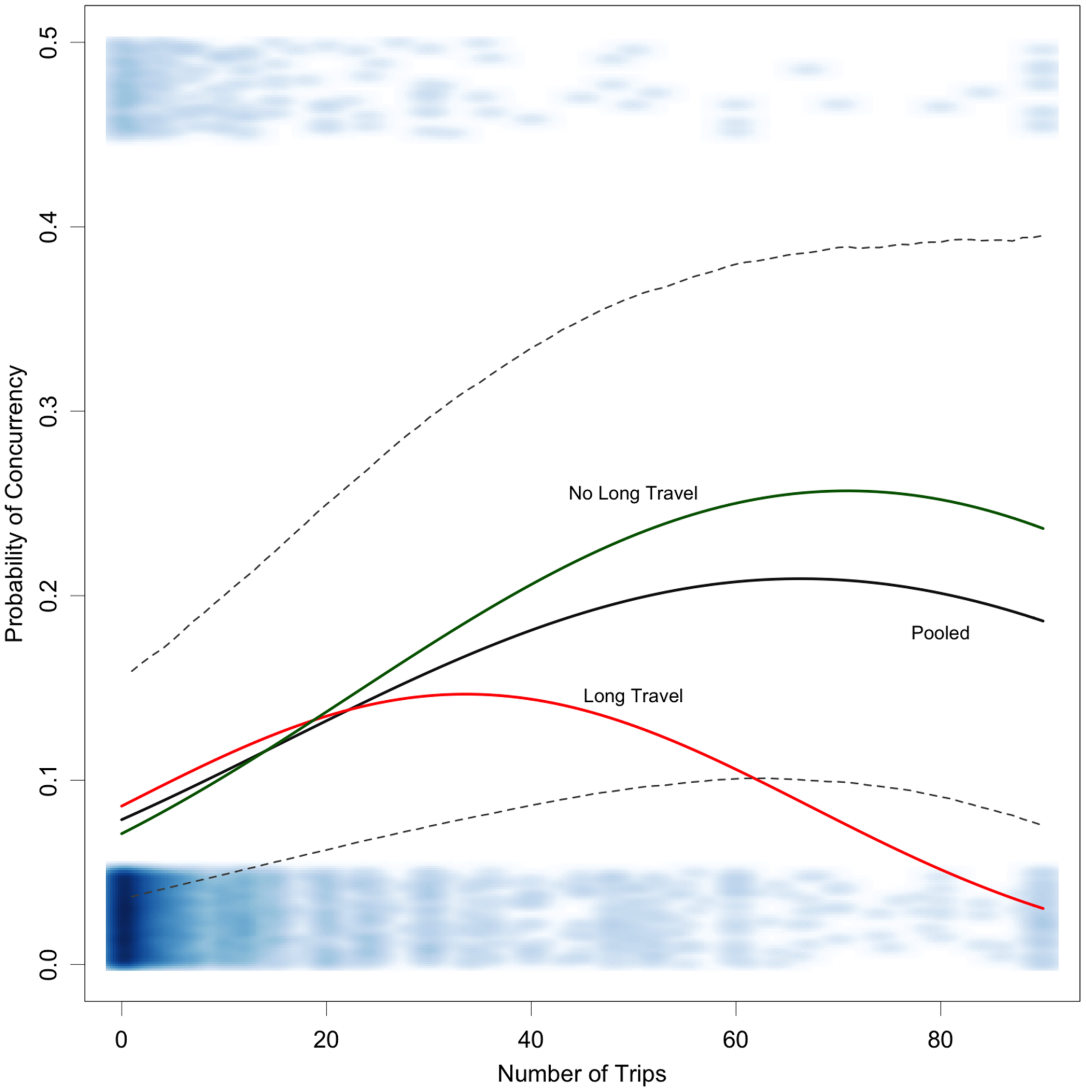
Relationship between number of overnight trips and the probability of any concurrent partnerships in the past year among men. The blue band at the bottom shows the data density by number of trips among those with partnership concurrency; the band at the top shows is the density for those who did report a concurrent partnership. Long travel represents men who reported travel of one month or longer in the last 12 months.

Because the coefficients for short-term mobility in the regression model were similar for polygamous and non-polygamous concurrency, we ran a combined analysis with a binary outcome of “any concurrency” and fit a logistic regression model with the same parameterization. Figure 1 shows the predicted probability of concurrency from both a quadratic interaction model fit and the fit of a pooled model without an interaction. In the pooled model, the probability of concurrency increases to a point (60 trips per year), and then decreases with each additional trip. In the interaction model, the effects of short-term mobility are stronger for those with no long-duration travel, compared to those with long-duration travel, starting at 20 trips (where the fitted lines cross).

Table 3 represents characteristics of partnerships. The vast majority of long-term cohabiting couples (>83%) had at least one member travel overnight within the past year, and in 41% of couples both partners traveled at least once overnight. Very few cohabiting couples reported that both partners traveled frequently (1.9%) or for a long duration (2.7%). In 14.6% of couples, only the male partner reported frequent travel, whereas only 7.3% of couples reported only the female partner frequently traveled. However, similar percentages of couples reported that only the female (11.8%) or only the male (11.9%) traveled for a long duration. Nearly 20% of the couples had at least one partner infected with HIV: 6.4% male only, 3.6% female only, and 9.6% both. One in ten couples reported any concurrency, almost entirely reported by the male partner, with 6% reporting non-polygamous concurrency only and 3% reporting polygamous concurrency.

**Table 3:** Descriptive Characteristics of Heterosexual Couples in Relationships ≥12 Months and Both Tested for HIV (n = 1,852) in the 2010–11 Zimbabwe DHS: Travel, HIV Serostatus, and Sexual Partner Concurrency at the partnership-level^1^.

	Neither		Male-Only		Female-Only		Both	
	%	95% CI	%	95% CI	%	95% CI	%	95% CI
**Short-term mobility**								
Overnight Travel^2^								
≥1 Trips	16.6	14.5–18.9	19.1	17.0–21.4	23.3	20.7–26.2	41.0	38.1–44.0
≥10 Trips	76.1	73.6–78.5	14.6	12.8–16.8	7.3	6.1–8.9	1.9	1.4–2.7
>1 Month Away	73.6	71.2–75.9	11.9	10.2–13.8	11.8	10.2–13.8	2.7	2.0–3.6
**Outcomes**								
HIV-Infected	80.3	78.2–82.3	6.4	5.4–7.7	3.6	2.9–4.6	9.6	8.3–11.1
Concurrency^2,3^	90.0	88.0–91.7	9.9	8.2–12.0	0.0	–	0.0	–

1 Analyses weighted with standard DHS sampling weights for study design and non-response.

2 In the past 12 months.

3 Of the 171 men who reported concurrent partners in the last 12 months, 57 had polygamous-only concurrency and 114 had additional non-wife or cohabitating partners in last 12 months.

Table 4 shows the results of the bivariate logistic regression mode testing whether short-term mobility by one or both partners in a couple was associated with partnership concurrency in the male partner. Both partners’ mobility and male-only frequent mobility was significantly associated with male non-polygamous concurrency (RR  = 2.07, 95% CI  = 1.38–3.10; RR  = 3.75, 95% CI  = 2.45–6.74). Female-only mobility was associated with less male non- polygamous concurrency (RR  = 0.53; 95% CI  = 0.29–0.95). No significant associations were seen with male polygamous concurrency and short-term mobility. Multivariate analyses of the dyad-level data were not performed due to small numbers in each of the subgroups.

**Table 4:** Association Between Dyadic Short-term mobility and Concurrency in the 2010–11 Zimbabwe DHS: Heterosexual Couples in Relationships ≥12 Months and Both Tested for HIV (n = 1,852).

	Concurrency			
	Male Non-Polygamous		Male Polygamous	
	RR	95% CI	RR	95% CI
**Short-term mobility**				
≥1 Trips				
Neither	1.00		1.00	
Male-only	0.93	0.54–1.60	1.59	0.78–3.26
Female-only	0.53	0.29–0.95	0.72	0.33–1.60
Both	2.07	1.38–3.10	1.24	0.66–2.32
≥10 Trips				
Neither	1.00		1.00	
Male-only	3.75	2.45–5.74	1.72	0.82–3.62
Female-only	0.68	0.28–1.65	0.81	0.27–2.40
Both^1^	1.35	0.35–5.20	–	
>1 Month Away				
Neither	1.00		1.00	
Male-only	1.04	0.58–1.84	1.43	0.56–3.66
Female-only	1.29	0.70–2.38	1.46	0.71–2.99
Both	1.98	0.77–5.10	1.01	0.14–7.52

1 Cells are undefined when no cases were observed.

## Discussion

Short-term mobility has the ability to impact population-level HIV transmission dynamics by facilitating partnership concurrency. Most research suggests that migrants, both short-term travelers and long-term permanent migrants, have a higher prevalence of HIV and sexual risk behaviors compared to non-migrants [10]. The risk propensity conceptual framework posits that mobility enables travelers to exhibit these underlying risk behaviors. We defined short-term mobility in a number of different ways, taking into account frequency and duration of travel, as well as short-term mobility as a partnership-level characteristic. In our analysis of the DHS data assessing adult men and women in Zimbabwe in 2010–11, we found several important links between short-term mobility and male partnership concurrency.

At the individual-level, there was strong evidence supporting our hypothesis of an association between short-term mobility and increased male concurrency. This may be indicative of concurrency facilitated through circular migration: an individual maintaining a partnership at home and adding a partner while away. We found that this relationship was non-linear: the increased risk of concurrency associated with travel declines with each additional trip at a threshold of about 5 trips per month. This may be due to competing time demands. While travelers are more likely to engage in sexual risk behavior when they are away from a primary partner due to freedom and anonymity [1], [10], very frequent travelers may behave differently than occasional travelers when away. Alternatively, the characteristics of travelers’ destinations for frequent versus infrequent travel may be associated with traveler’s risk behavior. Circular migrants or highly mobile individuals might have additional ‘main partners’ if they maintain additional residences; however, it is unlikely that they accumulate additional main partners in a linear fashion with each trip [41]. Additional longitudinal research on reasons for travel, behavior during travel, and characteristics of the sending and receiving communities is needed to address this non-linear association.

At the dyad level, there was evidence of a positive association between short-term mobility and male concurrency, but only when the male or both partners traveled. The probability of male concurrency was less when only women travel, possibly suggesting that males’ concurrency is driven by their own mobility and not by being left alone while their female partner travels. We had hypothesized that stay-at-home individuals with mobile partners would exhibit some increased concurrency, similar to findings that the sexual risk behavior of men in Tanzania increased when their wives traveled compared to when they themselves traveled [18]. However, the negative association we found is more consistent with prior research finding no difference in STI [8] or HIV [42] prevalence for partners of circular migrants. This may be due in part to social monitoring by the home community that suppresses potential increases in sexual risk behavior while the partner is away. We should note, however, that this finding does not conflict with the selection hypothesis, since the male was not selected as a migrant. Further dyadic research is needed to understand the mechanisms driving sexual risk and disease.

We were unable to test the direct association between female mobility in a couple and female concurrency, although women may also acquire HIV infection from outside partnerships [21], which might be due to travel. Vissers et al. found that women in Tanzania who lived in a different location than their spouses were more likely to exhibit risk behavior than cohabitating partners [22]. Additionally, a recent study in South Africa found that female migrants were more likely to be HIV-positive than female non-migrants, and that high risk sexual behavior increased the likelihood of HIV infection about twice as much in the context of migration for women [20]. However, most studies suggest that even if women do travel at relatively equal rates of men, they exhibit concurrency much less frequently. This may point towards sex-related differences in the risk propensity theory, wherein either the selection mechanism is weaker or the enabling mechanisms are attenuated due to the frequency, duration, or reasons for travel. Alternatively, female migrants may be just as likely as non-migrants to under-report concurrent partnerships as compared to men, a form of gendered social desirability bias.

Interestingly, we did not observe any significant associations between short-term mobility and polygamous concurrency among couples, and duration of travel was not statistically significant either. However, our findings do suggest that frequency and duration of travel are important to consider jointly: long-duration trips may be qualitatively different than multiple frequent trips, possibly due to differing reasons for travel or differing destination characteristics for long-term versus short-term frequent travelers.

Several studies have explored the relationship between migration or mobility and HIV risk specifically in Zimbabwe. A cohort study in rural Zimbabwe found no differences in sexual risk or HIV prevalence by migration status, which the investigators hypothesized may be attributable to the mature stage of the epidemic in which the disease has become more evenly geographically distributed [12]. However, the study withdrawal was non-trivial, and it is plausible that the non-informative censoring assumption may have been violated. Coffee et al. [23] used the baseline data from this study to examine both destination and duration of migration and mobility in rural Zimbabwe, finding that rural to urban migration is less important than rural to rural migration in spreading HIV. A prior analysis of the 1999 Zimbabwe DHS study data also found some differential effects in sexual risk behavior by the urbanity of the sending versus receiving area [29]. On the whole, this literature suggests that the somewhat varied outcomes may be attributable to the qualitative differences in reasons for and types of migration and mobility. Although we were unable to quantify the rural versus urban short-term mobility, our findings extend this line of research by estimating the joint effects of travel frequency and duration and also the differences between individual-level and dyadic risk.

We do not present analyses with HIV infection as the main outcome since we are unable to investigate the link between HIV and short-term mobility in the last year with incident HIV infection due to the cross-sectional study design. In fact, HIV infection could easily lead to travel if symptomatic HIV infection influences persons to migrate to obtain better treatment [4] or because of marital instability [43]. Infection-influenced travel may have important implications for further transmission dynamics [44]. Although the link between migration and HIV infection may be investigated with a prospective cohort study, the potential for study withdrawal related to migration seems inherently problematic [18]. The use of the detuned diagnostic assays could potentially be used, but are not currently within the DHS studies and the methods may be limited to antiretroviral-naïve populations [45].

This exploratory study of overnight travel and partnership concurrency has several limitations. New evidence suggests that the DHS consistently underestimates concurrency, especially for women [46]. The DHS does not ask whether relationships are ongoing at the time of the interview, so proxy measures must be calculated for recent partners, and our ability to construct correct measures of concurrency duration is limited. Our measure for concurrency risk is an oversimplification as well because the frequency of sexual activity within the main and secondary partnerships was not measured in the DHS. For example, it is not possible to determine whether the overall frequency of sex with the main partner remained constant upon the initiation of the relationship with the second partner, or whether the frequency with the main partner decreased (i.e., because the overall frequency across partners remained constant, a phenomenon called coital dilution) [40], [47]. The risk for forward transmission to the main partner would be less in the scenario with coital dilution, but would still be greater than if concurrency did not occur. We were also not able to measure condom use in each partnership. More broadly, there is potential for measurement error for sexual behaviors related to poor recall and social desirability, but its unclear whether this would be differential with respect to travel status; if not, our estimates are conservative. Future empirical research on concurrency should consider the possibility of coital dilution by measuring changes in sexual frequency over time. Qualitative research may also shed some light on some of the complex causal pathways hypothesized to link migration and mobility to sexual risk behavior and subsequently disease transmission.

Our measures of migration are limited as well. In the dyadic analysis, we have no information regarding if or when the couple traveled together. More importantly, we cannot align mobility episodes in time with concurrency episodes. Future work on travel, HIV, and sexual risk behavior should incorporate information on detailed characteristics of mobility, such as with whom traveled, the reason for travel, and destination of travel. There were other limitations to this analysis. First, this was primarily an exploratory study of the relationship between short-term mobility and partnership concurrency. Thus, we did not adjust the hypothesis test statistics for multiple comparisons, as the study was not statistically powered to generate inference on all the potential relationships here. There is a high probability of type I error in the inferential statistics presented.

In conclusion, we show evidence that short-term mobility is linked with increased male concurrency, and possibly in a pathway leading to increased HIV transmission at the dyadic- and population-level. This paper extends previous work by focusing specifically on sexual partnership concurrency and investigating the relationship between short-term mobility and concurrency at both the individual and partnership level. Future research should extend this analysis by further exploring the complex temporal relationships between these factors, and should consider whether to target travelers for HIV testing, treatment, or other social or behavioral interventions to prevent HIV.
